# Transcriptome profiling of flower buds of male-sterile lines provides new insights into male sterility mechanism in alfalfa

**DOI:** 10.1186/s12870-022-03581-1

**Published:** 2022-04-15

**Authors:** Bo Xu, Rina Wu, Fengling Shi, Cuiping Gao, Jia Wang

**Affiliations:** Key Laboratory of Grassland Resources of the Ministry of Education, College of Grassland Resources and Environment, Inner Mongolia Agricultural University, Hohhot, China

**Keywords:** Alfalfa, Ribosomal protein, Male sterility, Transcriptome, DEGs

## Abstract

**Background:**

The use of heterosis to produce hybrid seeds is a challenge to breeding for improved crop yield. In previous studies, we isolated a male sterile alfalfa hybrid and successfully obtained a genetically stable alfalfa male sterile line through backcrossing, henceforth named MS-4. In this study, we used RNA-seq technology to analyze the transcriptome profiles of the male sterile line (MS-4) and the male fertile line (MF) of alfalfa to elucidate the mechanism of male sterility.

**Results:**

We screened a total of 11,812 differentially expressed genes (DEGs) from both MS-4 and MF lines at three different stages of anther development. Gene Ontology (GO), and Kyoto Encyclopedia of Genes and Genomes (KEGG) analyses revealed that these DEGs are mainly involved in processes such as energy metabolism, lipid and amino acid metabolism, carbohydrate metabolism, in addition to cell synthesis and aging. The results from protein–protein interaction (PPI) network analysis showed that the ribosomal protein (*MS.Gene25178*) was the core gene in the network. We also found that transcriptional regulation was an influential factor in the development of anthers.

**Conclusions:**

Our findings provide new insights into understanding of the fertility changes in the male sterile (MS-4) of alfalfa.

**Supplementary Information:**

The online version contains supplementary material available at 10.1186/s12870-022-03581-1.

## Introduction

Heterosis or hybrid vigor is a natural phenomenon whereby the hybrid offspring of a genetically diverse cross outperform the parents in multiple traits, including yield, adaptability, and resistance to biotic and abiotic stresses. It is one of the most effective approaches for improving both yield and quality of crops [1, 2]. Using male sterile lines to achieve heterosis can effectively minimize self-degeneration, save the laborious steps of manual emasculation, and facilitate the harvesting of hybrid seeds. The use of male sterile lines to produce hybrid seeds has been widely tested and successfully applied to numerous crops [3, 4]. At the same time, male sterile lines are valuable materials in studying changes occurring during the development of stamens and anthers due to the interaction of cytoplasmic and nuclear genomes [5, 6].

Various conditions may lead to male sterility. Most of the male sterile lines were produced by impairment of anther formation or abnormal development. The development and formation of anthers is a complex process involving many metabolic pathways [7]. Metabolic pathways related to the development of anthers (that produce pollen) mainly include processes such as amino acid transport and metabolism [8], carbohydrate metabolism [9], lipid metabolism [10], energy conduction and metabolism [11]. Fang [12] reported reduced expression of glutamine synthetase in the amino acid biosynthesis pathway in cytoplasmic male sterile pepper, while Yang [7], detected significant differences in the expression of glycosyltransferase and glycosylhydrolase in the carbohydrate metabolism pathway between male fertile and male sterile lines of eggplant. Moreover, genes of carbon, starch, and sucrose metabolism were most abundant in the KEGG enrichment analysis. The *CaMF2* gene, which encodes a lipid transfer protein (LTP), is also considered to have an important function, where its silencing influenced pollen development in peppers [13]. For energy production and transformation, some enzymes and carrier proteins related to the mitochondrial respiratory chain are possibly related to male sterility in plants, and these have been explored by using the MS lines [14, 15]. Some genes involved in protein processing may also cause male sterility. The hub genes UbL40s (ubiquitin-60S ribosomal protein L40) and HSPs (heat shock protein) were found to be highly expressed at restrictive temperature (30 °C) and were suggested to be involved in fertility alteration in male sterile lines of rice [16].

Furthermore, the occurrence of male sterility is also related to abnormal tapetum development. The tapetum is the closest connection to the pollen mother cell (PMC) in the anther wall, providing nutrients for pollen development, which degrades later to release the microspores [17]. Therefore, the premature or delayed degradation of the tapetum can induce abnormal pollen development and could lead to male sterility [18]. Previous studies have connected specific genes to the tapetal programmed cell death (PCD) [19]. The mutant gene PTC2 (PERSISTENT TAPETAL CELL2) encodes the AT hook nucleus localization (AHL) protein in rice (*Oryza sativa*). This mutant exhibited defects in the pollen wall such as collapsed bacula and disordered tectum [20]. Yang [21] used the CRISPR/Cas9 system to obtain the male sterile mutant *osms1*. *OsMS1* encodes a plant homeodomain (PHD) finger protein, which acts as a transcriptional activator and regulates chorionic PCD in rice by interacting with tapered regulatory factors. Some transcription factors are also involved in the PCD of the tapetum and could play a vital role in the development of anthers [22, 23]. In Arabidopsis, the gene cascade DYT1-TDF1-AMS-MYB103-MS1 has been proposed to be involved in the development and timely degradation of tapetum and play an important role in regulating the complex changes associated with male fertility [24].

In previous studies, we isolated a male sterile alfalfa hybrid and successfully obtained a genetically stable alfalfa male sterile line through backcrossing, henceforth named MS-4 [25, 26]. Our previous research revealed that the pollen abortion of MS-4 mainly occurred during the mononuclear pollen formation period (flower bud length was 2 ~ 3 mm), resulting from the tapetal premature disintegration and creating shriveled and distorted mature pollen grains [27]. Since the mechanism of male sterility in the MS-4 line is not yet fully understood, we have not yet initiated commercial hybridization, as is common in other countries. Thus, there is an urgent need to conduct in-depth research to elucidate the mechanism of male sterility in the MS-4 line. This study used RNA-seq technology to perform comparative transcriptome analysis using alfalfa male sterile and male fertile lines. Our approach included the use of GO, KEGG pathways, PPI network analysis, and transcription factor family analysis to identify the gene networks and metabolic pathways that are related to male sterility in alfalfa. The results from this study will hopefully contribute to elucidating the mechanism of male sterility in alfalfa.

## Results

### An overview of the transcriptome data

A total of 18 libraries (2 genotypes × 3 developmental stages × 3 biological duplications) were sequenced, and a total of 293.01 Gb of clean data was obtained. A sum of 1,978,058,230 original reads was generated where each sample reached at least 14.43 Gb. The basic percentage values observed were: Q20 > 96.57%, Q30 > 90.37%, and the GC content > 42.2% (Supplementary Table 1). After filtering low-quality reads, a total of 1,956,318,172 clean reads was finally obtained. Clean reads from each sample were compared to the designated reference genome, and the comparison rate ranged from 86.14% to 90.38%. A total of 145,544 expressed genes was detected in this analysis, including 127,953 known genes and 17,591 new genes.

### Identification of differentially expressed genes

**Fig. 1:**
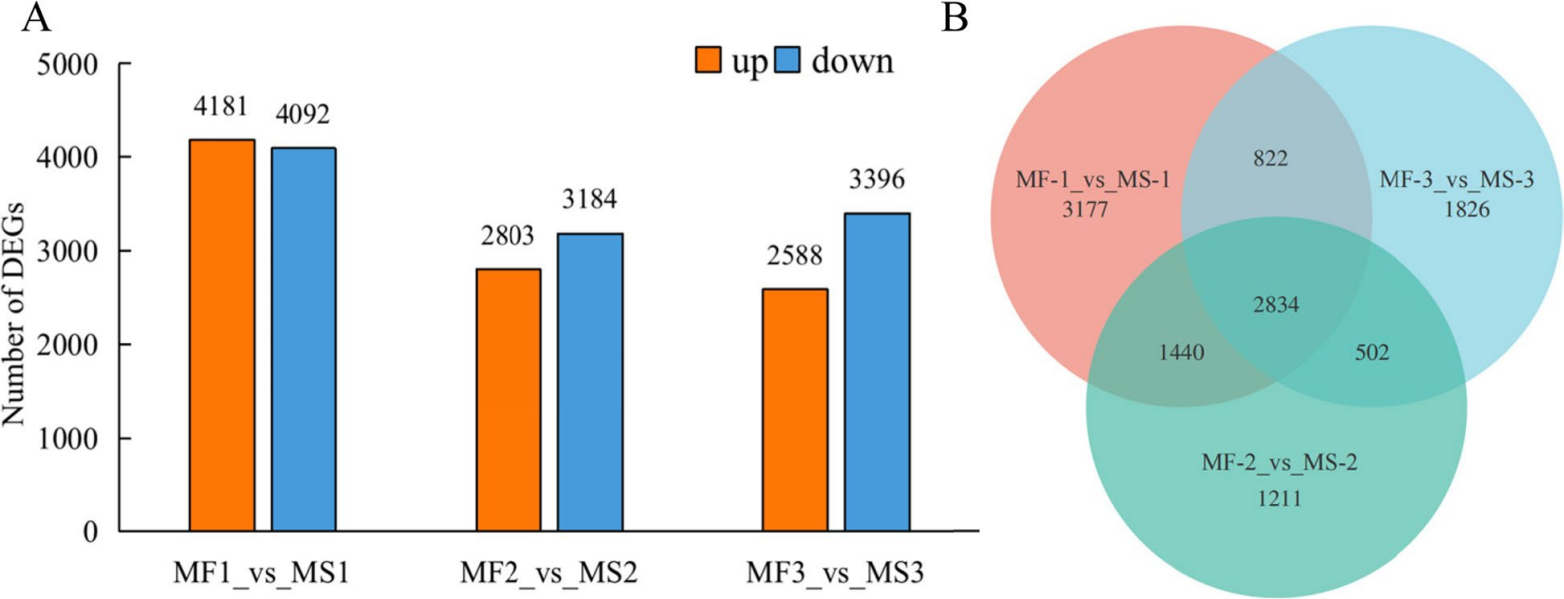
Statistics of DEGs. **A** The number of up- and down-regulated DEGs in three developmental stages. **B** Veen diagram analyses of differentially and stage-specific expression genes at three developmental stages

At three different stages of another development, a total of 11,812 genes was found to be differentially expressed between the flower buds of MS and MF plants. Among these differentially expressed genes (DEGs), 10,672 (52.72%) were down-regulated, and 9,572 (47.28%) were up-regulated (Fig. 1A). The number of DEGs at the first developmental stage was the largest (8,273), of which 4,181 were up-regulated, and 4,092 were down-regulated. At the second stage, there were more down-regulated genes than the up-regulated ones where 2,803 (46.82%) were up-regulated and 3,184 (53.18%) were down-regulated. At the third stage, the proportion of the down-regulated genes was higher (56.75%) and the number (3,396) was 31.22% higher than the number of up-regulated genes (2,588). Up to 2,834 DEGs were shared among the three developmental stages, while there were 3,177 most specifically expressed DEGs at the first stage (Fig. 1B). In summary, the 2,834 DEGs shared across the three developmental stages between MS-4 and MF lines may play a key role in another development.

### Hierarchical clustering analysis

**Fig. 2:**
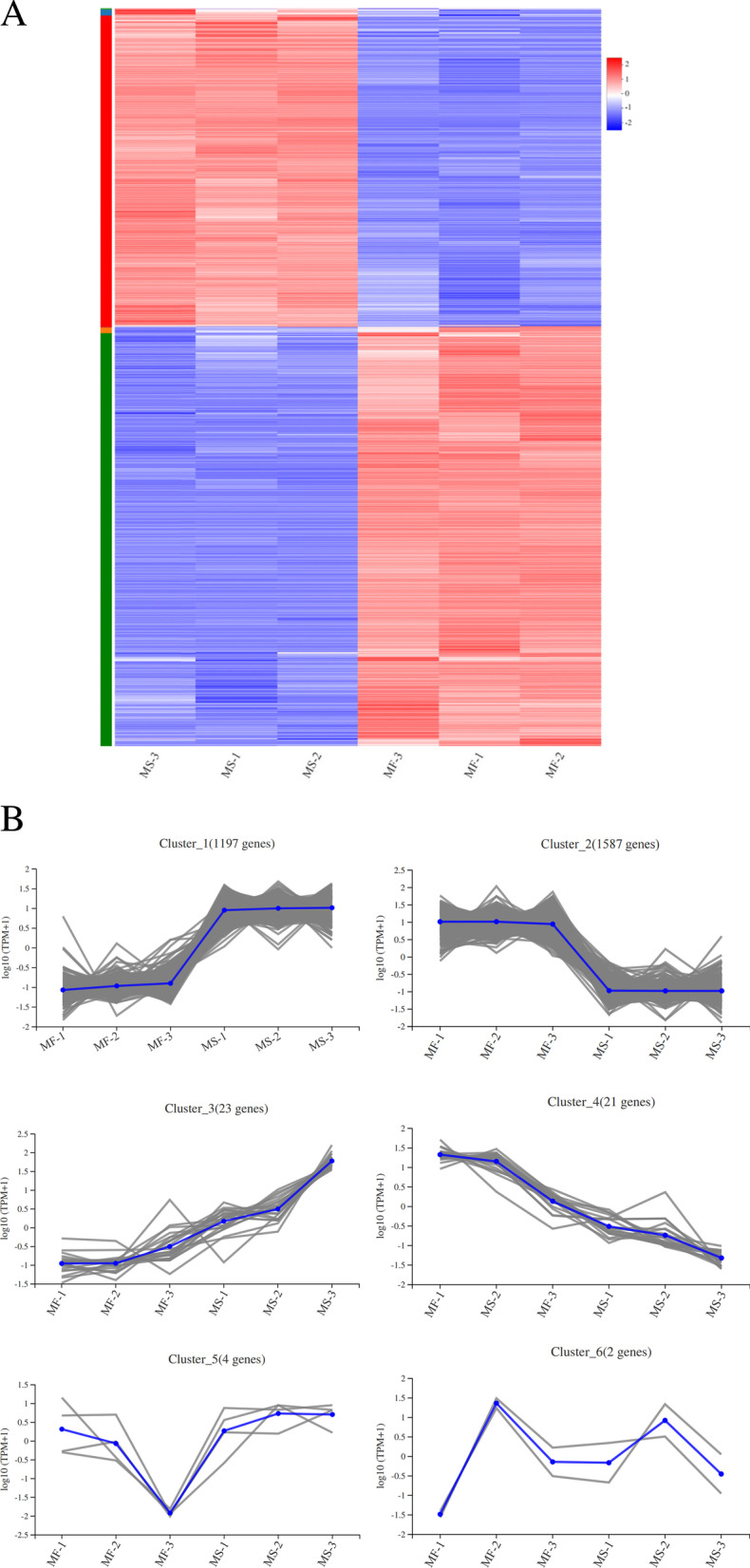
Clustering analysis of the 2,834 DEGs between the MF line and the MS-4 line. **A** Heat map diagram of the 2,834 DEGs. **B** Expression patterns of the 2,834 DEGs in the six clusters. The number of genes in each cluster was shown in parentheses

Based on the trend of gene expression under the same conditions, hierarchical clustering of 2,834 DEGs shared across the three developmental stages was conducted (Fig. 2A). The hierarchical clustering of gene expression profiles of MS-4 and MF lines at different developmental stages revealed that the gene expression patterns of these samples were different. The DEGs were subsequently divided into six clusters (Fig. 2B). Among them, cluster 1 and cluster 3 exhibited higher transcription levels in the male sterile line MS-4, with a total of 1,220 up-regulated DEGs. Cluster 2 and cluster 4 showed the opposite trend with a total of 1,608 DEGs suppressed in the male sterile line, MS-4. The number of DEGs showing down-regulated expression was more than those with up-regulated expression in the MS-4.

### Gene ontology and KEGG pathway analyses of DEGs

**Fig. 3:**
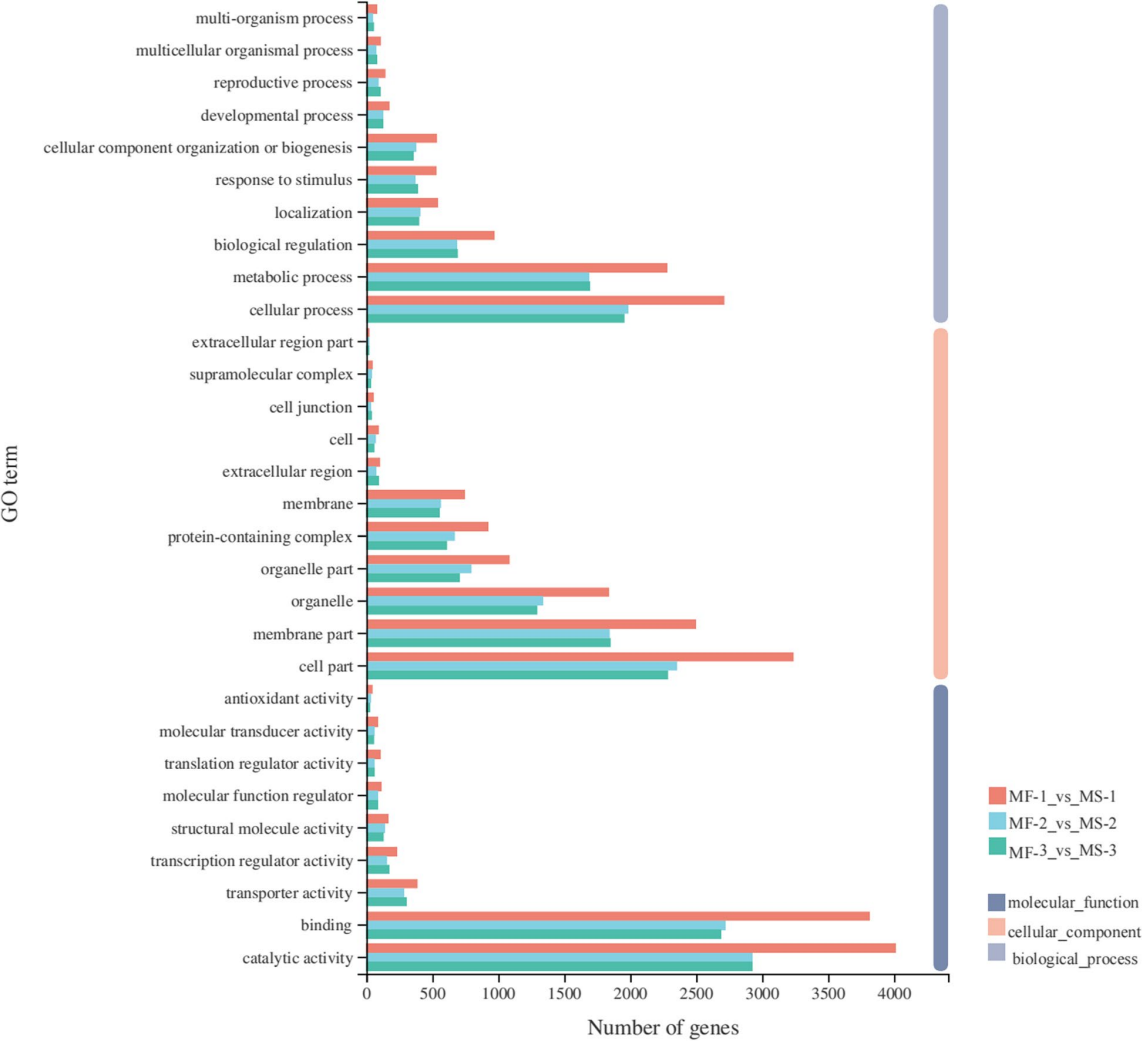
GO functional enrichment analysis of DEGs at the three stages of bud development

To understand the functional annotation of male fertility changes, GO annotation and enrichment analyses was performed on all DEGs at the three developmental stages (Fig. 3). The highly enriched terms of "Molecular Function" (MF) at the three stages of anther were binding (GO: 0,005,488) and catalytic activity (GO: 0,003,824). In the “cell component” (CC), these DEGs mainly participated in GO: 0,044,464 (cell part), GO: 0,044,425 (membrane part), GO: 0,044,422 (organelle part) and GO: 0,032,991 (protein-containing complex). In addition, "biological process" (BP) in terms of the cellular process (GO: 0,009,987), metabolic process (GO: 0,008,152), biological regulation (GO: 0,065,007), localization (GO: 0,051,179), response to stimulus (GO: 0,050,896) and cellular component organization or biogenesis (GO: 0,071,840) were highly enriched at all stages. The 2,834 DEGs shared at the three stages enriched the cellular process, cell part and catalytic activity (Supplementary Fig. 1).

**Fig. 4:**
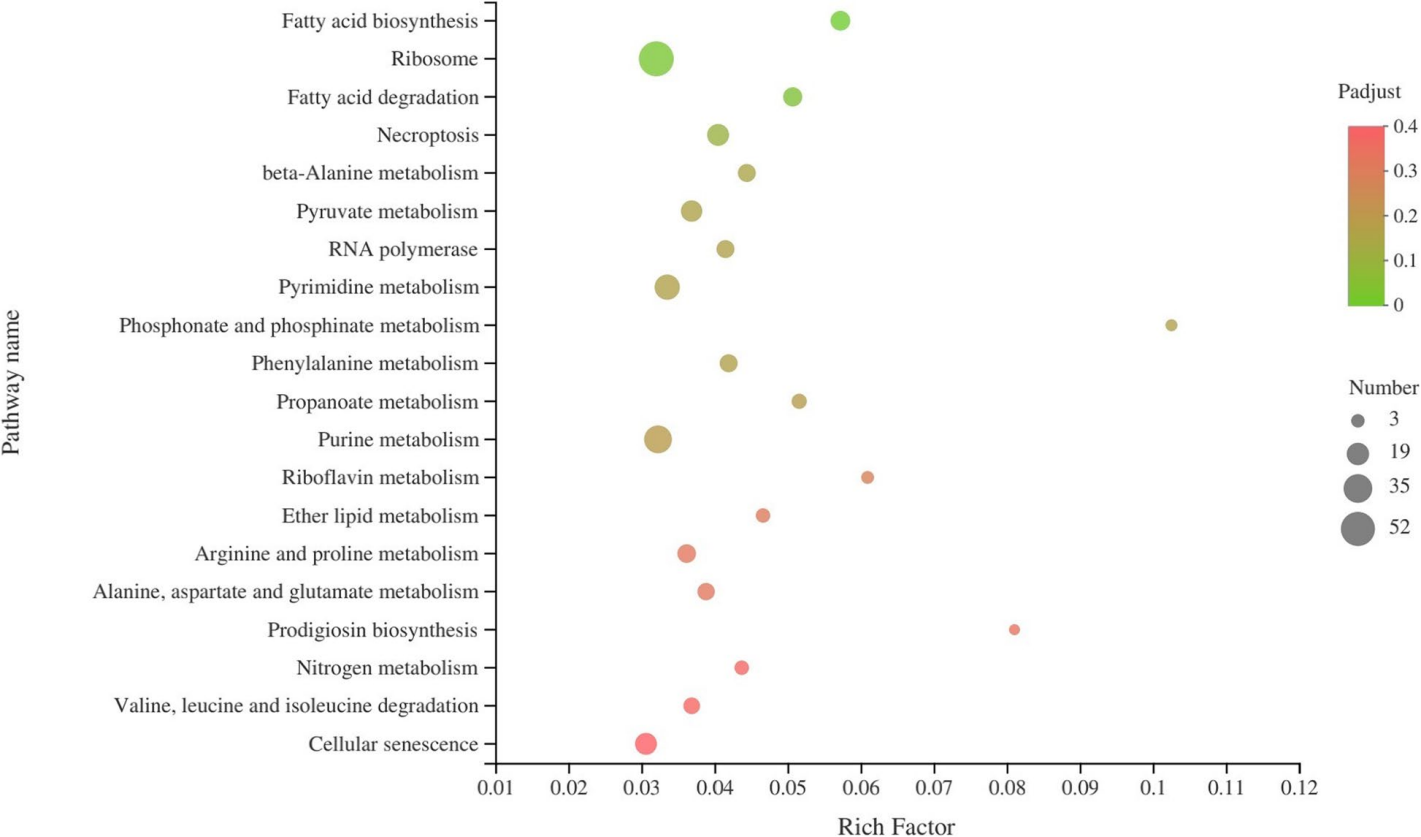
KEGG pathway enrichment analysis of the 2,834 DEGs between MF and MS-4

In order to determine the main biochemical and signal transduction pathways involved in DEGs, an enrichment analysis of the KEGG pathway was performed (Supplementary Fig. 2). A total of 2,834 DEGs was significantly enriched in ribosomal protein, fatty acid biosynthesis and degradation, pyrimidine and purine metabolism, cellular senescence, necroptosis, lipid and amino acid metabolism, and other pathways (Fig. 4). These annotations provided valuable information to determine the specific structure, function, process and pathway of the mechanism of pollen development in alfalfa.

### Protein–protein interaction network and topological analysis

**Fig. 5:**
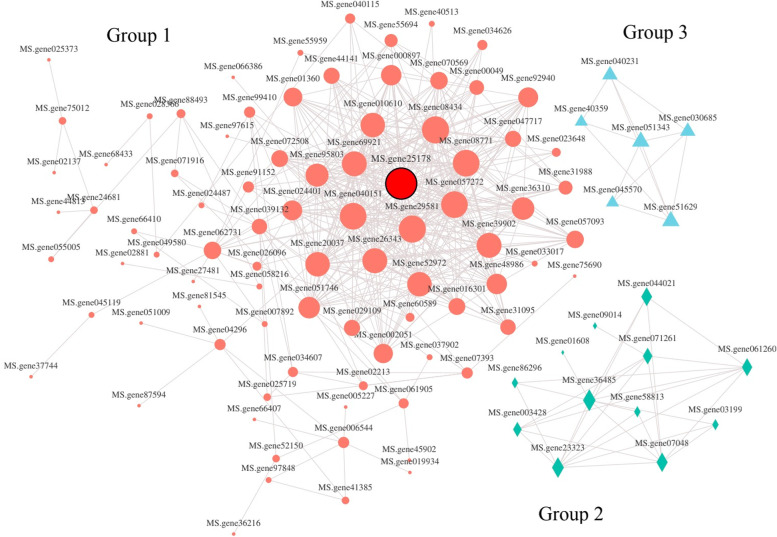
Co-expression network analysis of the partially predicted PPIs. Different colors and shapes represent different groups; the size of the nodes represents the importance in the network, the larger the node, the greater the importance of the gene in the network; the red-framed circle represents the hub gene

Protein–protein interaction (PPI) network and topological attribute analysis can identify important network relationships between proteins from complex biological data. To further understand the functional categories of 2,834 DEGs, a PPI network analysis was conducted for the top 500 ones with a comprehensive score of protein interactions. After removing the effect of free protein pairs (interacting proteins < 3), the PPI networks with 179 nodes and 500 edges were obtained, which mainly formed three networks with 84, 12 and 6 DEGs (Fig. 5, Supplementary Table 2). Most of the genes were assembled into Group 1, mainly with ribosomal protein family members, triose-phosphate transporter family protein (*MS.gene29581*) and aminoacyl- tRNA synthetase family (*MS.gene006544*, *MS.gene41385*) and others. These genes are involved in processes such as transport, translation and lipid and amino acid metabolism. Among them, ribosomal protein family members mainly included uS family (*MS.gene25178*, *MS.gene39902*, *MS.gene20037, MS.gene062731*), eS family (*MS.gene69921*, *MS.gene26343*, *MS.gene95803*, *MS.gene051746*), uL family (*MS.gene08434*, *MS.gene016301*, *MS.gene91152*). The genes in group 2 participate in DNA replication, including four DNA replication licensing factors that belong to the MCM family (*MS.gene36485*, *MS.gene23323*, *MS.gene07048*, *MS.gene061260*). In addition, the genes in group 3 are involved in the catalytic regulation process, including two peptidase T1B family members (*MS.gene040231* and *MS.gene40359*).

### Identification of the differentially expressed transcription factors

**Fig. 6:**
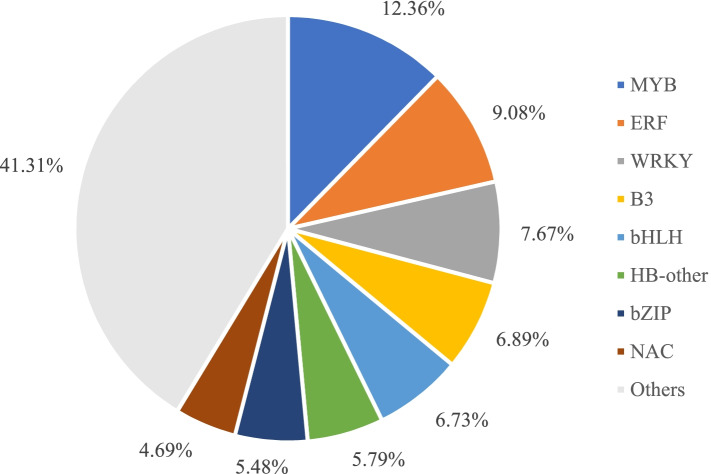
Distribution of differentially expressed transcription factors

The data showed that some DEGs belong to the genes encoding transcription factors (TFs). To better understand the molecular mechanism of male sterility, we analyzed the differentially expressed TFs. We identified 639 TFs from all DEGs. These TFs were divided into 40 families (Supplementary Table 3). The most abundant genes belonged to the MYB (12.36%), ERF (9.08%), WRKY (7.67%), B3 (6.89%), bHLH (6.73%), HB-other (5.79%), bZIP (5.48%) and NAC families (4.69%, Fig. 6). In addition, 132 TFs out of the 2,834 DEGs were shared in the three development stages (Supplementary Fig. 3). We speculate that these TFs form a highly complex transcription network and could play an important role in the mechanism of male sterility.

### Analysis of DEGs by qRT-PCR

**Fig. 7:**
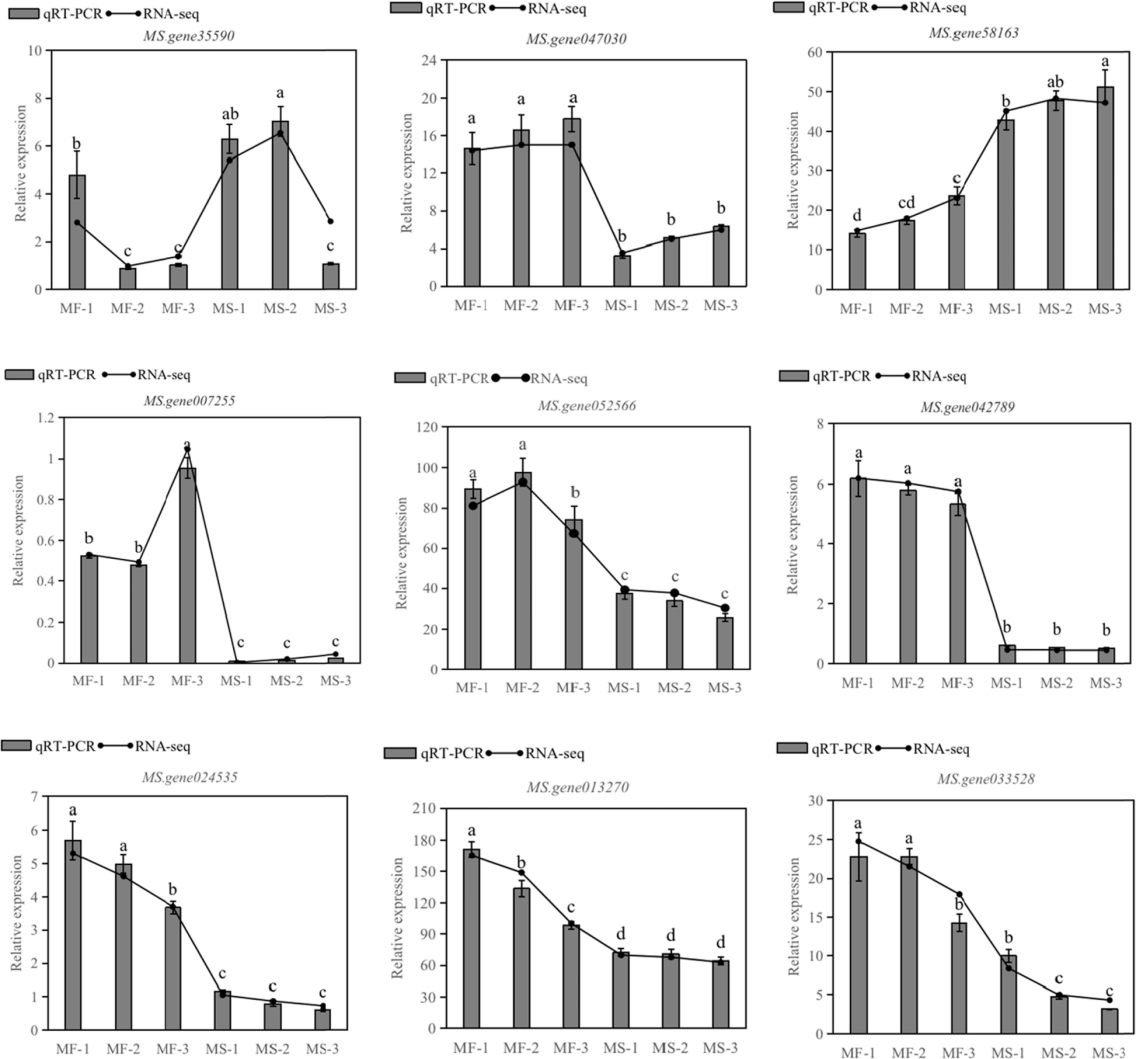
Verification of the expression of the selected DEGs was carried out via qRT-PCR. Error bars indicate the standard deviation of three biological replicates

According to RNA-seq data, the expression of nine selected genes was verified by qRT-PCR (Fig. 7). The genes included three genes related to tapetum and pollen development (*MS.gene35590*, *MS.gene047030*, *MS.gene58163*), ABCG39 (*MS.gene007255*), sugar transport protein (*MS.gene052566*), putative carboxylesterase (*MS.gene042789*), transcription factor MYB4 isoform X2 (*MS.gene024535*), NAC domain-containing protein 2 (*MS.gene013270*), and transcription factor bHLH18 (*MS.gene033528*). These genes exhibited similar expression trends to those obtained from the RNA-seq at the three stages of another development, confirming the reliability of RNA-seq analysis.

## Discussion

### Genes involved in tapetum and pollen development are related to male sterility

During male gametogenesis in flowering plants, the tapetum in the anther provides nutrients for the development and maturation of microspores and pollen walls [28]. Defective development of the tapetum was found to be responsible for male sterility in watermelon [29]. In this study, we identified 17 genes that were related to tapetum and pollen development (Supplementary Table 4). These genes were differentially expressed between the fertile and sterile lines. These differentially-expressed genes may have contributed to male sterility. The timely programmed cell death (PCD) of the tapetum is crucial where early or delayed start of PCD can cause male sterility [30, 31]. For example, SaNa-1A, a novel cytoplasmic male sterility (CMS) line of *Brassica napus*, was used to validate that abnormal tapetum degradation and anther development were the primary causes for anther abortion [32]. Previously, we observed that the tapetum layer of the sterile line MS-4 was prematurely disintegrated, and in this study, we identified six genes that are related to tapetum PCD. Furthermore, in the male sterility group, a gene labeled as male sterility (*MS.gene78221*) was down-regulated (Supplementary Fig. 4). These genes can be used as critical genes to test the effects of tapetum and pollen development on male sterility in alfalfa.

### Genes involved in carbohydrate and lipid metabolism are related to male sterility

Functional fertile pollen grains have a complete and complex outer wall structure [33], resulting from the joint action of multiple metabolic pathways and functional genes [34]. However, the mature pollen grains of MS-4 were shriveled and deformed. To explore the potential causes of these features, we performed enrichment analysis of, and annotated the DEGs at the three stages of bud development. The GO enrichment results of alfalfa anther transcriptome showed that DEGs related to biological processes were significantly enriched in "cellular processes", "metabolic processes" and "biological regulation". The KEGG pathway analysis of DEGs revealed that genes of "fatty acid biosynthesis", "lipid metabolism", "amino acid metabolism", "pyrimidine and purine metabolism", and "cellular senescence", were among those significantly enriched. These genes are involved in energy metabolism, lipid and amino acid metabolism, carbohydrate metabolism, and some cell synthesis and aging processes. Carbon pathways provide energy and carbohydrates for plant growth and development [35], while glutamine plays a central role in pollen development and amino acid metabolism [36]. Fatty acids (FA) regulate the homeostasis of reactive oxygen species (ROS), and dysfunction of *FAX1* can destroy oxygen free radicals, which could contribute to male sterility [34]. Changes in the expression level of genes in these pathways may influence fertility. In our study, one carbohydrate metabolism pathway DEG (*MS.gene052566*) and one lipid metabolism pathway DEG (*MS.gene042789*) were selected for RT-PCR analysis to compare their expression levels in the male sterile and fertile (Fig. 7). The data showed that the expression levels of the three genes were lower in MS-4 than in the MF line. Therefore, we speculated that reducing carbohydrate and lipid metabolism affected the transmission of energy and nutrients, leading to male sterility.

### ABCG transporters involved in altering the fertility

As an important component of the process of transport of nutrients and energy across the cell membranes, the ABC transporters play a key role in another development [37]. The ABCG transporters constitute the largest subfamily of ABC transporters [38], which have been reported to be involved in the development of anther or pollen (pollen wall). Zhou et al. [39]. speculated that β-ketoacyl-[acyl carrier protein] synthase I (*SiKASI*) was crucial for fatty acid metabolism and might interact with *ABCG18* causing abnormal pollen development in *Arabidopsis*. The rice male sterile mutant *osabcg26* exhibited defects in tapetal cells, lipid Ubisch bodies and pollen exine, possibly due to a disturbance in the homeostasis of anther lipid metabolism and transport [40]. Moreover, other plant ABCG transporters such as *AtABCG1*, *AtABCG16* [41], *AtABCG9*, *AtABCG31* [42], *OsABCG3* [43], and *OsABCG15* have been reported to be involved in the development of pollen or anther development in *Arabidopsis* and *Oryza sativa* [44]. In this study, according to RT-PCR results, a gene labeled as ABC transporter G family member 39 (ABCG39, *MS.gene007255*) was down-regulated in the male sterile group. This suggests that ABCG39 (*MS.gene007255*) may participate in the regulation of anther development to ultimately cause male sterility. However, this hypothesis needs further experiments to be verified.

### Genes related to ribosomal proteins are involved in male sterility

Cytoplasmic male sterility (CMS) in plants is caused by genetic conflicts or communication barriers between nuclear and mitochondrial genes [45]. The genes of CMS are typically new chimeric regions genes, and most of these genes are known to encode ribosome and mitochondrial electron transport chain complexes [46]. Additionally, ribosomal protein content is an indicator of mitochondrial function, where some studies have shown that ribosomal proteins were closely related to fertility changes. Lower content of ribosomal protein during anther abortion was observed in cotton, suggesting that insufficient synthesis of ribosomal protein may have hindered pollen production [47]. However, Zhao et al. [48] found that the 60S ribosomal protein L13a-4-like was up-regulated in the male sterile line and hence was related to the abortion of microspores in cotton. These results indicate that the up- or down-regulation of some key ribosomal proteins may lead to male sterility. Sunok [49] and Ding et al. [50] speculated that ribosomal proteins may participate in tapetum PCD and could play an important regulatory role in tapetum development. The results from the PPI network analysis of this study indicate interactions between ribosomal protein family members, triose-phosphate transporter protein family, and aminoacyl-tRNA synthetase family. Topological analysis (Fig. 5) indicated that the 40S ribosomal protein S2 (*MS.gene25178*) was the core gene in the network and was up-regulated in the male sterile line (MS-4). The up-regulated expression of this gene in the present study (Supplementary Fig. 5), consistently with that of Zhao's on cotton, suggests a critical role for 40S ribosomal proteins in regulating male sterility in alfalfa. These results also indicate that ribosomal protein genes could also induce fertility changes, and future research should focus on in-depth analysis of these genes.

### The TF genes are related to male sterility

Many studies on male sterility have shown that TFs play an important role in plant growth and anther development [51, 52]. The helix-loop-helix transcription factor (bHLH) has been reported to regulate the development of the tapetum [53, 54]. *OsbHLH138* was identified as a key gene that regulates thermo-sensitive male sterility in rice [55]. Moreover, *MYB80* (formerly *MYB103*) can delay PCD by activating transcription of the UNDEAD gene that results in male sterility [56]. These results illustrate the importance of bHLH transcription factors. Han et al. [57] obtained several male sterile lines in which the MYB transcription factor *OsMS188* was knocked out in rice, in which *OsMS188* was verified as the key regulator of tapetum development and sporopollenin synthesis. Furthermore, Cheng et al. [28] suggested that NAC transcription factor was a central gene related to another development and may cause male sterility in cotton. In this study, we found that 639 genes belonged to 40 families of TFs in all DEGs, and that some TFs belonging to the same family were differentially up- or down-regulated. These results are consistent with recent studies on bud dormancy in the grapevine [58], suggesting that members of the same TF family may play different roles in the development of pollen in alfalfa, forming a more complex transcriptional regulatory network. We selected two DEGs annotated as transcription factors (transcription factor MYB4, *MS.gene024535*; and transcription factor bHLH18, *MS.gene033528*) for RT-PCR analysis (Fig. 7). The data showed that the expression of the two genes in MS-4 was reduced (relative to the MF line). These results indicate that TFs play an essential role in regulating male sterility.

## Conclusions

Transcriptomic analysis of anthers at three different developmental stages in the male sterile (MS-4) of alfalfa as compared to the male fertile (MF) line revealed that the underlying mechanism of male sterility is complex. Male sterility in alfalfa is a network process involving many biological processes and pathways, including catalytic activity, cell component, cellular processes, ribosomal proteins, fatty acid biosynthesis, and metabolic function. The findings from this study provide several insights to elucidate the mechanism of male sterility based on changes observed from anther development in the male sterile line, MS-4 of alfalfa. In addition, this study also indicated that the 40S ribosomal protein S2 (*MS.gene25178*) plays an important role in inducing male sterility. Further research is needed on the key metabolic pathways and candidate genes to define the mechanism of male sterility in alfalfa.

## Material and methods

### Plant materials

We isolated a natural sterile mutant of *Medicago varia Martin*. cv. 'Caoyuan No. 1', and we obtained a stable male sterile line MS-4 from it after a few years of cultivation. The tapetum layer of the sterile line MS-4 was prematurely disintegrated, and the mature pollen grains were shriveled and deformed. MS-4 plants were grown under normal conditions in the fields on the Inner Mongolia Agricultural University campus in China. In our previous research, we conducted cytological studies on MS-4 and the male fertile (MF) plant and found that the pollen abortion observed with MS-4 mainly occurred during mononuclear pollen formation [59]. Therefore, flower buds from MS-4 and MF plants were collected at the three stages of anther development: pollen mother cell formation stage (bud length 0.8 ~ 2 mm), mononuclear pollen formation stage (bud length 2 ~ 3 mm), and pollen maturity stage (bud length 4 ~ 5 mm). Flower buds of the same plant at the same developmental stage were peeled off by removing the calyx and were flash frozen before being stored (-80 °C) for subsequent transcriptome sequencing. Each sample at each developmental stage included three biological replicates, which were sequenced separately.

### RNA extraction, sequencing, and RNA-seq analysis

Trizol (TaKaRa, Dalian, China) was used to extract total RNA from alfalfa anther tissues, and DNase I (Takara, Japan) was used to remove genomic DNA. The RNA library was established using TruSeq™ RNA sample preparation Kit (Illumina, San Diego, CA). SuperScript double-stranded cDNA synthesis kit (Invitrogen, CA) was used with six-base random primers (Illumina) to synthesize one-strand cDNA using mRNA as template, and then to perform two-strand synthesis to form a stable double-stranded structure. After the cDNA was enriched by PCR, a 200–300 bp band was recovered using 2% agarose gel. After being quantified with TBS380 (Picogreen), the library was sequenced with an Illumina HiSeq Xten/NovaSeq 6000 sequencing platform for high-throughput sequencing, with a sequencing read length of PE 150. Complete transcriptome analysis of 18 libraries was conducted. Reference gene source used was from:

https://figshare.com/projects/whole_genome_sequencing_and_assembly_of_Medicago_sativa/66380 [60].

### Identification and bioinformatic analysis of DEGs

DESeq2 software based on negative binomial distribution was used to analyze raw counts, and *p*-adjust < 0.05 and |log2^FC^|≥ 1 (the expression difference was more than twice) were used as the criteria for selecting significant differential expression of genes. Bioinformatic analyses of DEGs were carried out on the Meiji Bio-Cloud Platform (https://www.i-sanger.com/). GO (http://www.geneontology.org/) used Fisher method for exact test, and BH was the multiple test correction method. When the corrected *p* value (*p*adjust) < 0.05 was applied, it was considered that genes of GO function and KEGG pathways (http://www.genome.jp/kegg/) [61, 62] were significantly enriched [63]. The DEGs of protein–protein interaction (PPI) network were predicted by STRING [64] (https://string-db.org/), and the drawn network diagram and network node degree were analyzed by Cytoscape 3.5.1. Transcription factors (TFs) were identified using PlantTFDB 4.0 (http://planttfdb.cbi.pku.edu.cn/).

### qRT-PCR verification

UEIris II First-Strand cDNA Synthesis Kit (Code NO. R2028 US EVERBRIGHT INC, Suzhou, China) was used to reverse transcribe RNA. Fast Super EvaGreen qPCR Master Mix kit (Code NO. S2008 US EVERBRIGHT INC, Suzhou, China) was used, which contained a 20 μL solution (50 ng cDNA as a template; 10 × ROX reference dye), for qRT-PCR in a LightCycler 480 (Roche). This was conducted on each sample. The amplification protocol was as follows: 95 °C for 2 min, then 40 cycles at 95 °C for 10 s, 56 °C for 1 min and 72 °C for 10 s, and then melt curve at 95 °C 15 s, 60 °C 1 min, and 95 °C 15 s. Supplementary Table 5 lists the sequence of the specific primers used for qRT-PCR. *β*-Actin was used as an internal control. All qRT-PCR analyses were performed in three replicates. The relative gene expression levels were calculated using the 2^−ΔΔCt^ method [65]. The relative gene expression levels were analyzed via analysis of variance (ANOVA, *p* < 0.05).

## Supplementary Information

**Additional file1:** **Table S1.** An overview of the transcriptome data.

**Additional file2:** **Fig. S1.** GO functional enrichment analysis of the 2,834 DEGs.

**Additional file3:** **Fig. S2.** Pathway classification statistics histogram of all DEGs.

**Additional file4:** **Table S2.** PPI network information.

**Additional file5:** **Table S3.** Statistics of transcription factor families.

**Additional file6:** **Fig. S3.** Transcription factors distribution among 2,834 DEGs.

**Additional file7:** **Table S4.** 17 genes related to tapetum and pollen development.

**Additional file8:** **Fig. S4.** Relative expression of MS.gene78221 via qRT-PCR.

**Additional file9:** **Fig. S5.** Relative expression of MS.gene25178 via qRT-PCR.

**Additional file10:** **Table S5.** Primer information.

## Data Availability

Datasets are available at NCBI project PRJNA751239. The transcriptome datasets are available in the NCBI Short Read Archive (SRA) database with the accession number: SAMN20512309, SAMN20512310, SAMN20512311, SAMN20512312, SAMN20512313, SAMN20512314, SAMN20512315, SAMN20512316, SAMN20512317, SAMN20512318, SAMN20512319, SAMN20512320, SAMN20512321, SAMN20512322, SAMN20512323, SAMN20512324, SAMN20512325, SAMN20512326.

## Abbreviations

MS-4: Male sterile line
DEGs: Differentially expressed genes
GO: Gene Ontology
KEGG: Kyoto Encyclopedia of Genes and Genomes
PPI: Protein–protein interaction
LTP: Lipid transfer protein
PMC: Pollen mother cell
PCD: Programmed cell death
PTC2: Persistent tapetal cell2
AHL: AT hook nucleus localization
PHD: Plant homeodomain
TFs: Transcription factors
BP: Biological process
MF: Molecular function
CC: Cell component
CMS: Cytoplasmic male sterility
FA: Fatty acids
ROS: Reactive oxygen species
bHLH: Helix-loop-helix transcription factor
UbL40: Ubiquitin-60S ribosomal protein L40
HSP: Heat shock protein
