# Supplementary figures and images for: Transcriptome profiling of flower buds of male-sterile lines provides new insights into male sterility mechanism in alfalfa
Source: BMC Plant Biol. 2022 Apr 15;22:199. doi: 10.1186/s12870-022-03581-1 (PMC9013074; doi:10.1186/s12870-022-03581-1)

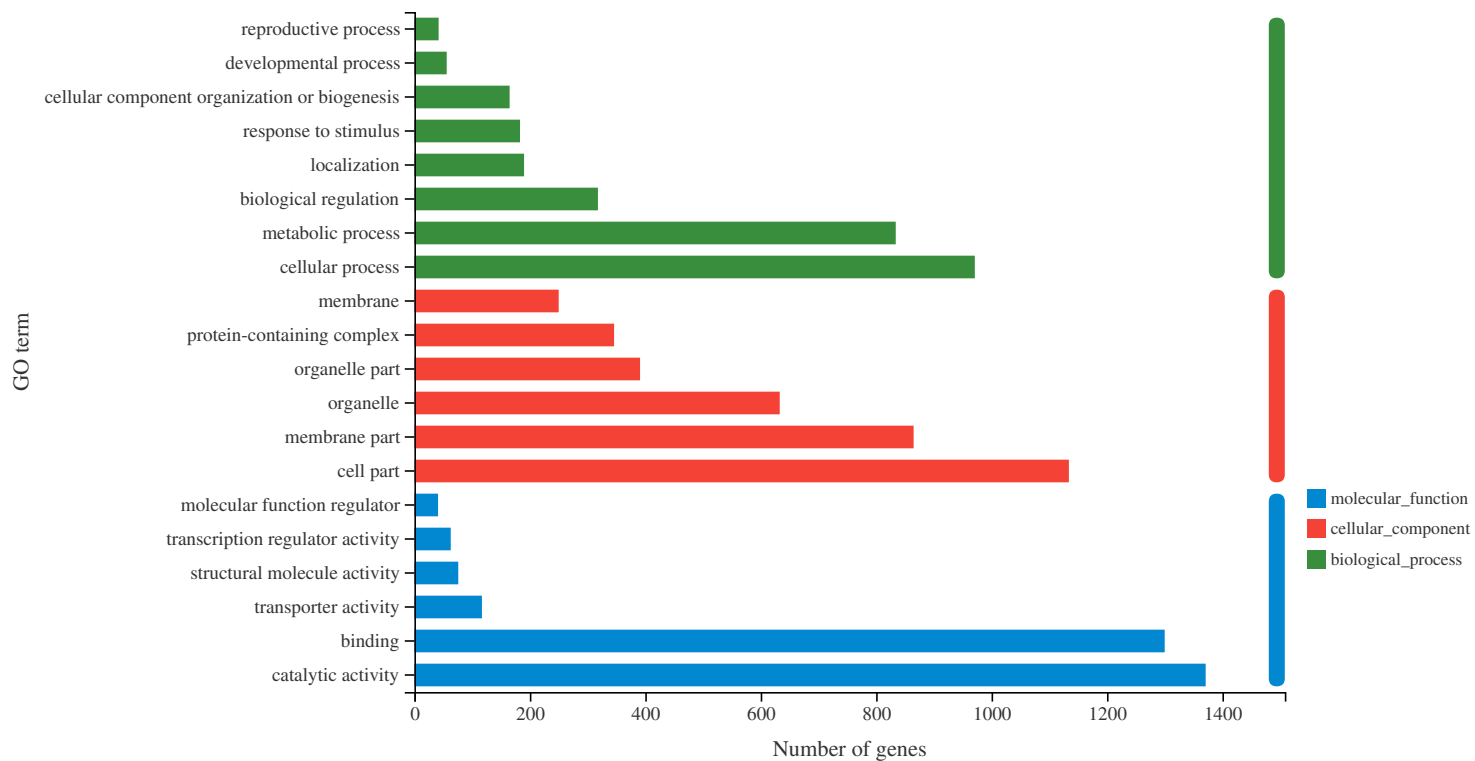

Supplement: Supplementary file 2 — Additional file2: Fig. S1. GO functional enrichment analysis of the 2,834 DEGs. [file 12870_2022_3581_MOESM2_ESM.pdf]

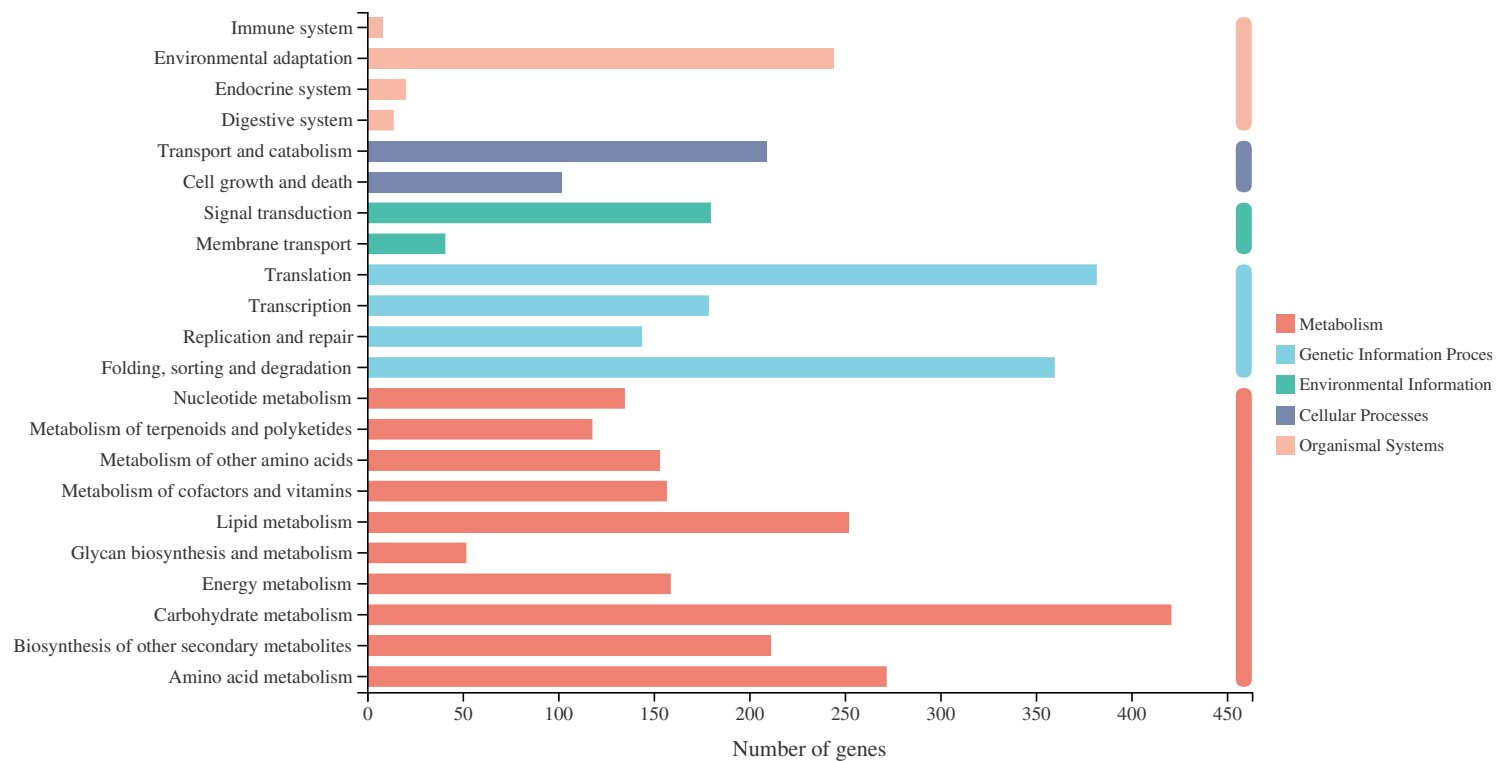

Supplement: Supplementary file 3 — Additional file3: Fig. S2. Pathway classification statistics histogram of all DEGs. [file 12870_2022_3581_MOESM3_ESM.pdf]

Statistics of TF family

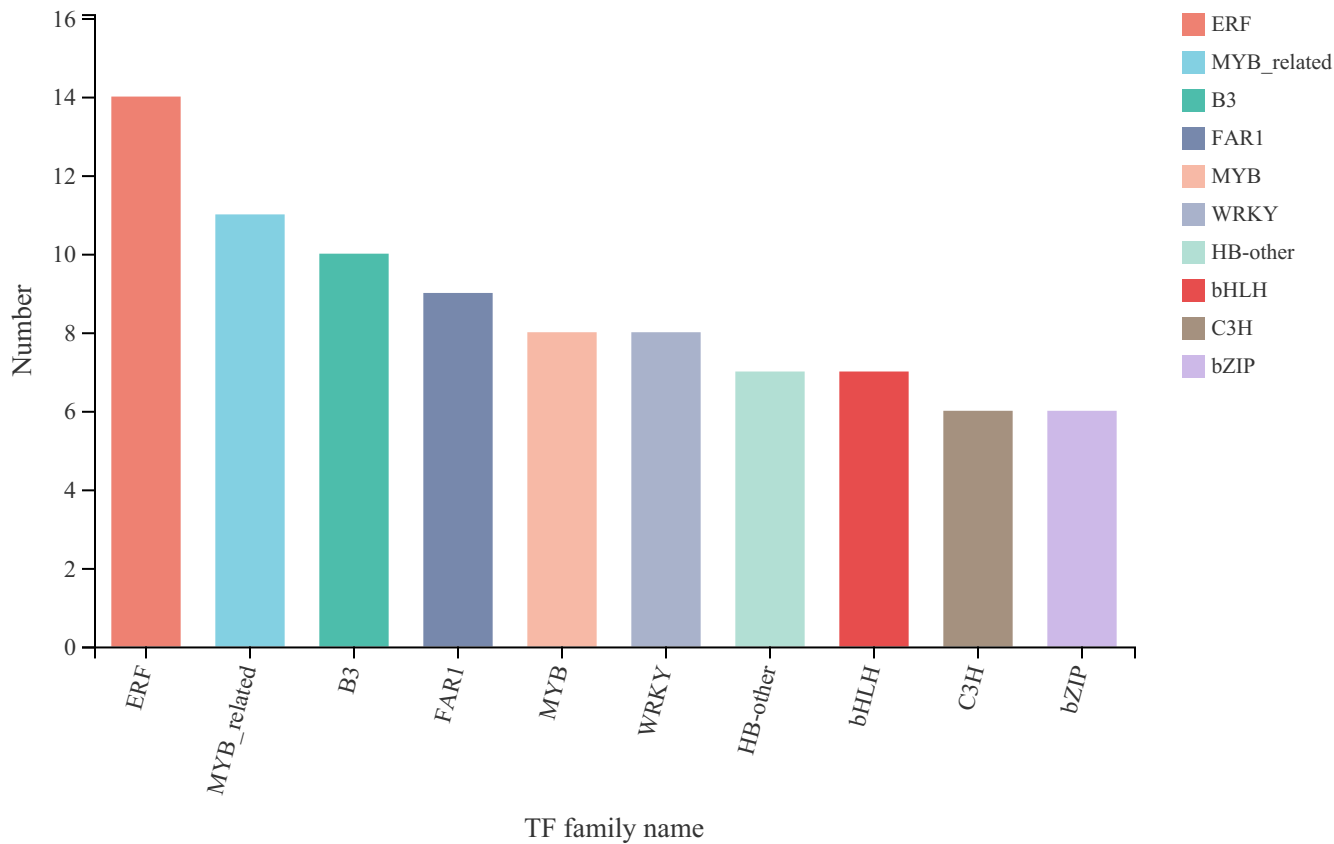

Supplement: Supplementary file 6 — Additional file6: Fig. S3. Transcription factors distribution among 2,834 DEGs. [file 12870_2022_3581_MOESM6_ESM.pdf]

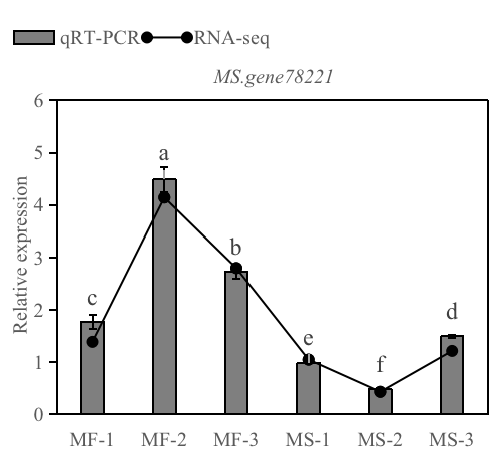

Supplement: Supplementary file 8 — Additional file8: Fig. S4. Relative expression of MS.gene78221 via qRT-PCR. [file 12870_2022_3581_MOESM8_ESM.png]

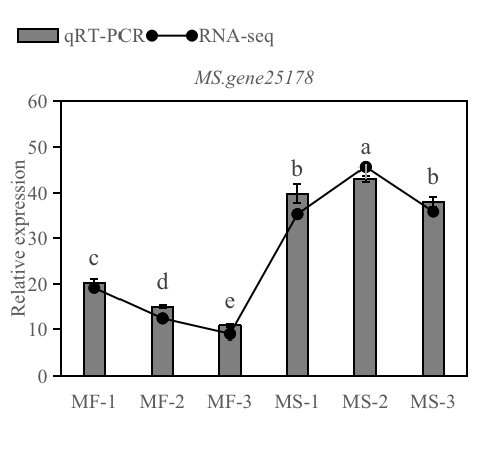

Supplement: Supplementary file 9 — Additional file9: Fig. S5. Relative expression of MS.gene25178 via qRT-PCR. [file 12870_2022_3581_MOESM9_ESM.png]
